# Secretory pathway retention of mutant prion protein induces p38-MAPK activation and lethal disease in mice

**DOI:** 10.1038/srep24970

**Published:** 2016-04-27

**Authors:** Berta Puig, Hermann C. Altmeppen, Sarah Ulbrich, Luise Linsenmeier, Susanne Krasemann, Karima Chakroun, Claudia Y. Acevedo-Morantes, Holger Wille, Jörg Tatzelt, Markus Glatzel

**Affiliations:** 1 Institute of Neuropathology, University Medical Center Hamburg-Eppendorf, Hamburg 20246, Germany; 2 Department of Biochemistry of Neurodegenerative Diseases, Institute of Biochemistry und Pathobiochemistry, Ruhr University Bochum, Bochum 44801, Germany; 3 Centre for Prions and Protein Folding Diseases and Department of Biochemistry, University of Alberta, Edmonton, T6G 2M8 Alberta, Canada

## Abstract

Misfolding of proteins in the biosynthetic pathway in neurons may cause disturbed protein homeostasis and neurodegeneration. The prion protein (PrP^C^) is a GPI-anchored protein that resides at the plasma membrane and may be misfolded to PrP^Sc^ leading to prion diseases. We show that a deletion in the C-terminal domain of PrP^C^ (PrPΔ214–229) leads to partial retention in the secretory pathway causing a fatal neurodegenerative disease in mice that is partially rescued by co-expression of PrP^C^. Transgenic (Tg(PrPΔ214–229)) mice show extensive neuronal loss in hippocampus and cerebellum and activation of p38-MAPK. In cell culture under stress conditions, PrPΔ214–229 accumulates in the Golgi apparatus possibly representing transit to the *R*apid *E*R *S*tress-induced *E*xpor*T* (RESET) pathway together with p38-MAPK activation. Here we describe a novel pathway linking retention of a GPI-anchored protein in the early secretory pathway to p38-MAPK activation and a neurodegenerative phenotype in transgenic mice.

Secretory or plasma membrane bound proteins are translated, folded and post-translationally modified in the endoplasmic reticulum (ER) and Golgi. The ER exerts quality control early in the secretory pathway^1^ and, therefore, many (but not all) folding-defective proteins are retained in the ER, translocated to the cytosol, polyubiquitinated and targeted to the ubiquitin proteasome system (UPS) for ER-associated degradation (ERAD) (reviewed in^2^,^3^). Overload of misfolded proteins challenges the ER capacity, leading to ER stress and induction of the unfolded protein response (UPR) to restore homeostasis (reviewed in^4^,^5^). When this stress becomes chronic, it results in cell death. In protein misfolding disorders, such as Alzheimer’s or prion diseases, chronic ER stress may contribute to neurodegeneration^6^,^7^,^8^.

The prion protein (PrP^C^), a glycosylphosphatidylinositol (GPI)-anchored membrane protein targeted to detergent resistant membranes (DRMs)^9^, plays fundamental roles in prion diseases, a group of fatal neurodegenerative disorders^10^,^11^,^12^. In humans, they can be sporadic (e.g. sporadic Creutzfeldt-Jakob disease (sCJD)), genetic (e.g. Gerstman-Sträussler-Scheinker syndrome (GSS) or Fatal Familial Insomnia (FFI)) or acquired (iatrogenic CJD (iCJD) or new variant CJD (vCJD)). A key event in the pathogenesis of prion diseases is the misfolding of PrP^C^ to PrP^Sc^, biochemically characterized by its insolubility and partial resistance towards proteinase K digestion.

PrP variants that lack the GPI-anchor are mainly degraded by ERAD^13^,^14^,^15^,^16^,^17^, while GPI-anchored variants of PrP containing mutations within the globular C-terminal domain are not amenable to ERAD^18^. Instead, GPI-anchored mutants of PrP, both artificially constructed mutants and naturally occurring disease mutants, undergo RESET (*R*apid *E*R *S*tress-induced *E*xpor*T*) pathway prior degradation^19^. RESET is a stress-inducible pathway by which diverse misfolded GPI-anchored proteins dissociate from ER-resident chaperones, bind to ER export factors and traffic to the Golgi. After RESET, they transiently appear on the plasma membrane before being endocytosed for lysosomal degradation.

We have studied a C-terminal deletion mutant of PrP^C^ (PrPΔ214–229) in neuronal cell lines and transgenic mice. We expected our mutant to cause a clear phenotype for two main reasons. On the one hand, point mutations at alpha helices 2 and 3 (α2 and α3) lead to genetic prion disease^13^,^14^,^15^ and its total deletion in mice leads to intraneuronal PrP aggregates^20^. On the other hand, shedding of PrP^C^ occurs between Gly228 and Arg229 by the sheddase ADAM10 and substrate recognition is achieved by a steric motive^21^,^22^. Shedding of PrP^C^ helps to maintain PrP^C^ homeostasis at the plasma membrane and shed forms of PrP^C^, found in CSF and blood, hold important functions. Thus, alterations in the recognition site of ADAM10-mediated shedding^23^,^24^ may lead to absence of presumably neuroprotective functions of shed PrP^C^^25^,^26^.

PrPΔ214–229 is GPI-anchored and characterized by an immature glycosylation status. In cell culture, PrPΔ214–229 is partially retained in the ER and under stress conditions, it reaches the trans-Golgi network (TGN) where it is retained, suggestive of a RESET pathway that does not undergo subsequent degradation to the lysosomes. Transgenic mice expressing PrPΔ214–229 (Tg(PrPΔ214–229)) present with neurodegeneration and a fatal neurological disease with increased p38-MAPK phosphorylation. Activation of p38-MAPK is also seen in cell culture under stress conditions. Thus, we show that retention in the secretory pathway of a misfolded GPI-anchored protein can be associated with a specific signaling pathway, leading to neurodegeneration in mice, with potential implications in human prion diseases.

## Results

### The C-terminal deletion (Δ214-229) in PrP^C^ interferes with maturation in the secretory pathway

**Figure 1:**
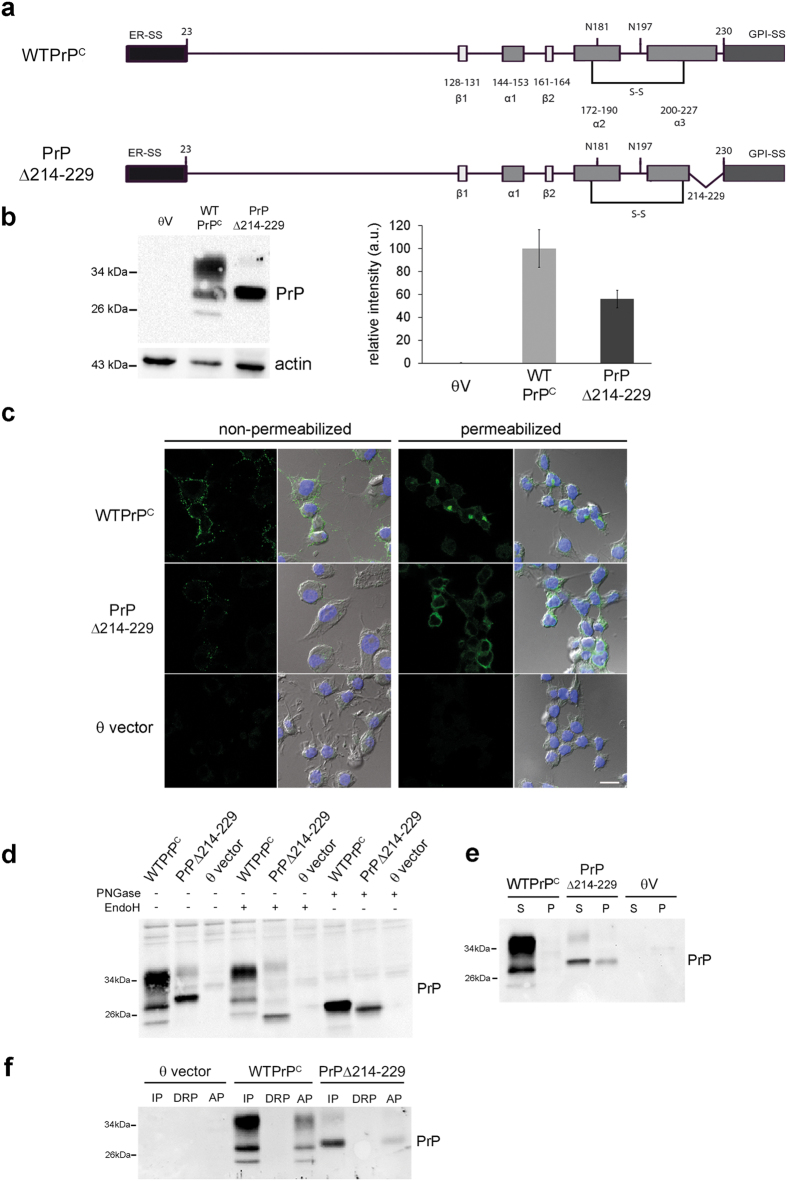
Characterization of WTPrP^C^ and PrPΔ214-229 in stably transfected N2a cells. (**a**) Schematic illustration of the constructs used in the study (mouse sequence). ER-SS: ER signal sequence; α 1–3: α-helices; β1, 2: β strands; S-S: disulfide bond; N181 and N196: N-linked oligosaccharide chains; GPI-SS: signal sequence for GPI-anchor attachment. In PrPΔ214–229 the deletion between residues 214–229 is shown. (b) Western blot developed with the 3F4 antibody. PrPΔ214–229 expresses mainly an immature band at around 29 kDa. θV are cells transfected with empty vector. The graphic shows the quantification of expression levels where the amount of WTPrP^C^ is set to 100%. Error bars indicate standard error of the mean (SEM). (c) Representative immunofluorescence of PrP (detected with 3F4 antibody; green) under non-permeabilizing and permeabilizing conditions. Under non-permeabilizing conditions it can be observed that less PrPΔ214–229 reaches the plasma membrane compared to WTPrP^C^. Under permeabilizing conditions PrPΔ214–229 shows diffuse intracellular staining. Scale bar is 10 μm. (d) Treatment of stably transfected N2a cells with EndoH shows that, as expected, WTPrP^C^ is completely resistant to enzymatic digestion whereas PrPΔ214–229 is sensitive. Treatment with PNGase F shows that in both constructs bands decrease in size and are brought to the size of the unglycosylated isoform. (e) After treating cell lysates with Triton X-100 and separating the supernatant (S) from the pellet (P) by centrifugation, PrPΔ214–229 is found in the pellet (P) fraction, indicative of aggregation. (f) Triton X-114 phase separation assay. IP: insoluble pellet where GPI-anchor proteins are mainly found; DRP: detergent-resistant phase; AP: soluble phase. Note that PrPΔ214–229 is also found in the IP fraction, indicating GPI-anchoring.

It has been described that misfolded GPI-anchored PrP follows an alternative ERAD pathway for degradation^18^,^19^. We have deleted 16 C-terminal amino acids (PrPΔ214–229) leaving the disulfide bond (amino acid 178–213) and the GPI-anchor attachment site (omega site, Ser230) intact (Fig. 1a). We generated mouse neuroblastoma cells (N2a) stably expressing wild-type PrP^C^ (WTPrP^C^) or PrPΔ214–229. The presence of the 3F4 tag allowed us to discriminate between endogenous PrP^C^ and transfected constructs. Cells transfected with PrPΔ214–229 expressed a main band at around 29 kDa when assessed by Western blot (Fig. 1b) in a proportion about ~60% of the amount of WTPrP^C^. Confocal microscopy showed plasma membrane localization (non-permeabilized cells) and perinuclear localization (permeabilized cells) for WTPrP^C^. PrPΔ214–229 showed reduced expression at the plasma membrane and diffuse intracellular staining (Fig. 1c). Digestion of cell lysates with Endoglycosidase H (EndoH, cleaving immature mannose-rich oligosaccharides) and N-Glycosidase F (PNGase F, removing all N-linked glycans) as well as a Triton X-114 assay^27^ confirmed presence of non-complex glycans and GPI-anchorage for PrPΔ214–229 (Fig. 1d,e). To study solubility of PrPΔ214–229, samples were treated with Triton X-100 and separated in pellet (insoluble) and supernatant (soluble) fractions by centrifugation. PrPΔ214–229 was present in the pellet fraction indicative of misfolding (Fig. 1f). In order to exclude a cell type bias, we also investigated transfected neuroblastoma SHSY5Y cells (presenting negligible levels of endogenous PrP^C^). As in N2a cells, PrPΔ214–229 in SHSY5Y cells was EndoH sensitive (Supplementary Fig. 1a), partially insoluble (Supplementary Fig. 1b) and mainly resided in intracellular compartments (Supplementary Fig. 1c).

**Figure 2:**
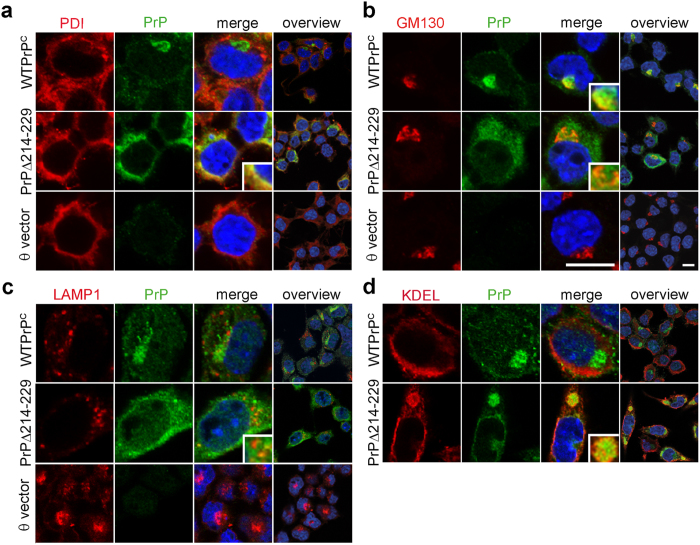
PrPΔ214–229 is mainly retained in the ER in N2a cells. (**a**) Confocal microscopy of double immunocytochemistry for PrP (detected with 3F4 antibody; green) with PDI (ER marker; red) or (**b**) GM130 (Golgi marker; red). Whereas WTPrP^C^ mainly colocalizes with GM130, PrPΔ214–229 colocalizes with PDI, although some degree of colocalization is seen with GM130 (**b** inset). Scale bar is 10 μm. (**c**) Confocal immunocytochemistry for PrP and the lysosomal marker LAMP1 (red). In few instances a partial colocalization with LAMP1 is seen for PrPΔ214–229 (c, inset). (**d**) Labeling of KDEL chaperones (in red) colocalizing with PrPΔ214–229 (detected with 3F4 antibody; green).

Co-staining with protein markers of ER (PDI), Golgi (GM130) and lysosomes (LAMP1) showed co-localization of WTPrP^C^ with the Golgi apparatus and the plasma membrane as described^28^, whereas PrPΔ214–229 was mainly found in the ER (Fig. 2a). Only rarely could we observe lysosomal localization of WTPrP^C^ or PrPΔ214–229 (Fig. 2c), mainly in cells with high PrPΔ214–229 expression (Fig. 2c, inset). Interestingly, clusters of ER-resident chaperones containing the KDEL motif colocalized with PrPΔ214–229 (Fig. 2d) implying a response to improper folding.

Taken together, we could show that PrPΔ214–229 in neuronal cells is GPI-anchored, immaturely glycosylated and mainly retained in the ER.

### Expression of PrPΔ214–229 leads to a fatal neurological disease in mice

**Figure 3:**
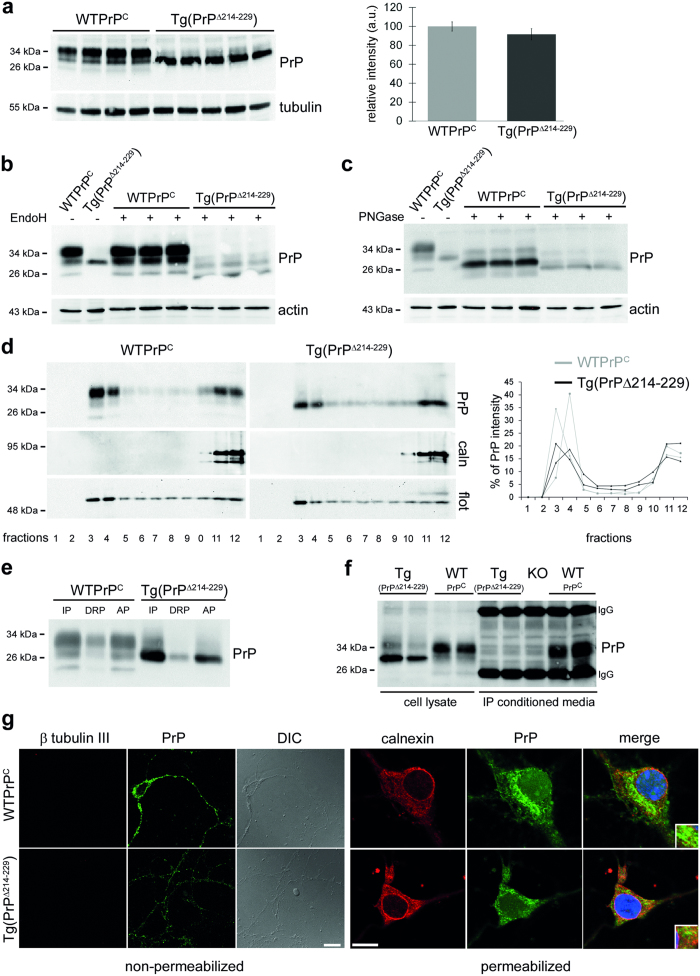
Characterization of transgenic PrPΔ214–229 mice (Tg(PrPΔ214–229)). (**a**) Representative Western blot (detected with POM1 antibody) of frontal cortex brain homogenates. PrPΔ214–229 presents a main band at ~29 kDa with a faint upper band. The graphic shows quantification of expression levels. The amount of WTPrP^C^ is set to 100%. Error bars indicate SEM. (**b**) Brain homogenates treated with EndoH. PrPΔ214–229 is largely EndoH sensitive although a minor fraction is non-sensitive (note the presence of an upper glycosylated band). (**c**) Treatment with PNGase F digests PrP to the size of the non-glycosylated isoform in both Tg(WTPrP^C^) and Tg(PrPΔ214–229) brain homogenates. (**d**) DRMs isolation. Both, WTPrP^C^ and PrPΔ214–229 are mainly found in fractions 3 and 4 (as shown in the graph on the right) indicating presence in lipid raft. Flotillin (flot) is a lipid raft marker and calnexin (caln) is a non-lipid raft fraction marker. (**e**) Triton X-114 phase separation assay. IP: insoluble pellet where GPI-anchor proteins are mainly found; DRP: detergent-resistant phase; AP: soluble (aqueous) phase. PrPΔ214–229 is found in the IP fraction indicating GPI-anchoring. (**f**) Analysis of PrP shedding in primary neurons. Shed PrP was immunoprecipitated (IP) from conditioned media using POM2 antibody and assessed in parallel with cell lysates by Western Blot. Primary neurons from *Prnp* knock-out mice (KO) served as negative control. No shed PrP was detected in the conditioned media of Tg(PrPΔ214–229) neurons. IgG: capturing antibody bands. (**g**) Confocal microscopy of PrP (POM1 antibody; green) in hippocampal primary neurons under non-permeabilizing and permeabilizing conditions. Under non-permeabilizing conditions, PrPΔ214–229 can only partially reach the plasma membrane. β tubulin III (red) proves lack of intracellular staining. DIC is phase contrast. Under permeabilizing conditions, PrPΔ214–229 presents a diffuse staining partially colocalizing with calnexin (red) used as an ER marker whereas PrP^C^, although also partially colocalizing with calnexin, seems to be mainly present in another organelle presumably Golgi, as observed in N2a cells (Fig. 2). Scale bar is 10 μm.

To study the consequences of the ER retention *in vivo* we generated transgenic mice expressing PrPΔ214–229. Two lines of transgenic mice were generated on a PrP^C^ knock-out background, with one line showing low expression (Supplementary Fig. 2) and one line expressing physiological levels of PrPΔ214-229 (Fig. 3a). We observed the three banding pattern for PrP^C^ in control mice (WTPrP^C^), whereas Tg(PrPΔ214–229) mice showed a prominent band at ~29 kDa and a very weak band at ~35 kDa (Fig. 3a). After EndoH or PNGaseF digestion of brain homogenates (Fig. 3a–c), we observed EndoH sensitive glycans in Tg(PrPΔ214–229) mice, suggesting lack of conversion into complex structures, as observed with N2a and SHSY5Y cells. Of note, a very faint fraction of PrPΔ214–229 showed complex glycosylation suggesting that a subset can be further processed in the secretory pathway. After Triton X-114 phase separation assay, PrPΔ214–229 from brain homogenates was mainly present in the insoluble pellet (IP) suggesting GPI-anchorage (Fig. 3e). Moreover, after solubilization with Triton X-100 and sucrose gradient ultracentrifugation, it mainly localized to DRMs (Fig. 3d), further demonstrating GPI-anchorage. Immunocytochemistry of primary neurons under non-permeabilizing conditions showed reduced plasma membrane levels of PrPΔ214–229 (Fig. 3g). Under permeabilizing conditions, a diffuse intracellular staining is observed for PrPΔ214–229, colocalizing with the ER marker calnexin (Fig. 3g). No proteolytically shed PrPΔ214–229 was detected in conditioned media of primary neurons (Fig. 3f) indicating that PrPΔ214–229 is mainly retained at the secretory pathway and, as a consequence, ADAM10-mediated shedding is absent.

**Figure 4:**
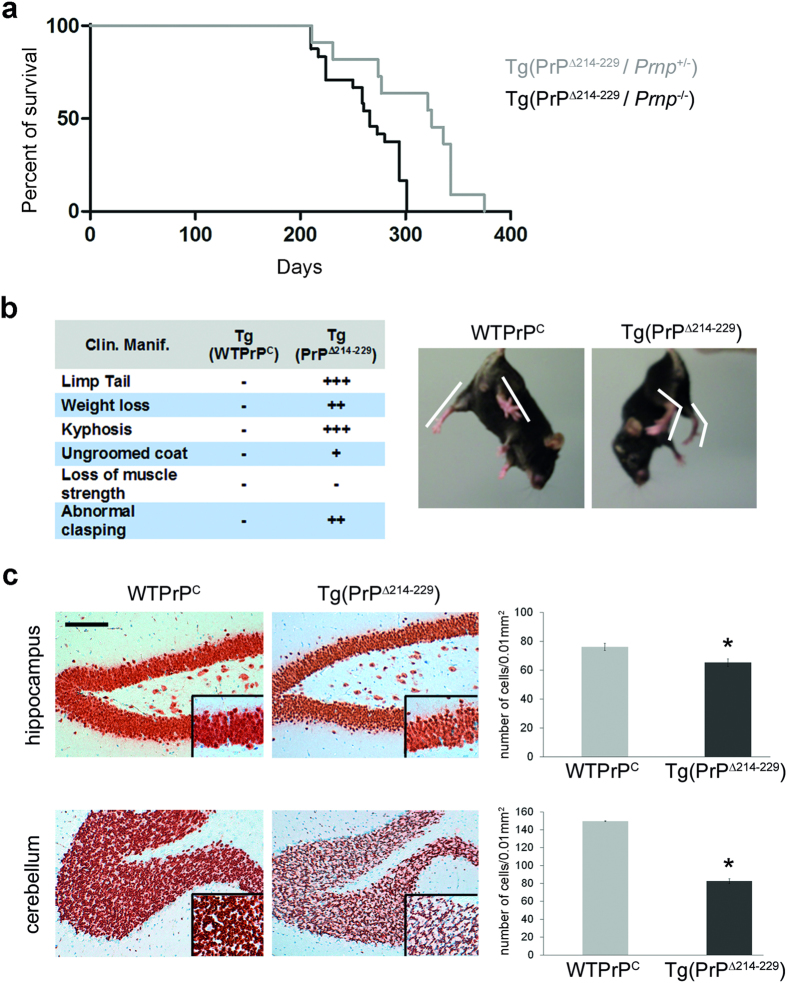
Tg(PrPΔ214–229) mice present with a neurological disease and die around 38 weeks of age with loss of hippocampal and cerebellar granular neurons. (**a**) Kaplan-Meier survival curve of Tg(PrPΔ214–229) mice (black, n = 21) and Tg(PrPΔ214–229) mice backcrossed with C57BL/6 wild-type mice expressing one allele of PrP^C^ (Tg(PrPΔ214–229/PrP^+/−^) (grey, n = 10). There is a significant delay to terminal stage of disease in Tg(PrPΔ214-229/PrP^+/−^) mice. ***p < 0,001 (*p*-values are given in the main text). (**b**) Clinical manifestations of Tg(PrPΔ214–229) mice. As shown in the pictures, Tg(PrPΔ214–229) mice present with absence of clasping but presence of an abnormal leg spreading when lifted at the tail. (**c**) Representative pictures of NeuN staining in cerebellum and hippocampus. Neuronal loss in Tg(PrPΔ214–229) mice is mainly observed in the granular cell layer and in the hippocampal CA1 layer. Quantifications show number of cells/0.01 mm^2^ for hippocampal CA1 layer and cerebellar granular layer (*t*-test: *p < 0.05). Error bars represent SEM.

Interestingly, Tg(PrPΔ214–229) mice developed a progressive fatal neurological disease with a mean survival time of 270.28 ± 6.47 days (Fig. 4a,b; Supplementary Fig. 3a). Genetic reintroduction of one allele of wild-type *Prnp* partially rescued the phenotype and prolonged survival to 315 ± 15.12 days (Fig. 4a; Breslow (generalized Wilcoxon) test (^*^p = 0.01)) arguing in favor of PrP-dependent effects. Neuropathologically, a significant neuronal loss in the cerebellar granular layer (Student’s *t*-test, *p* = 0.044) and the CA1 region of the hippocampus (Fig. 4c; *t*-test *p* = 0.002) with discrete spongiosis in white matter and no obvious astro- or microgliosis was seen (Supplementary Fig. 3b).

### Activation of p38-MAPK correlates with neurodegeneration and disease in Tg(PrPΔ214–229) mice

**Figure 5:**
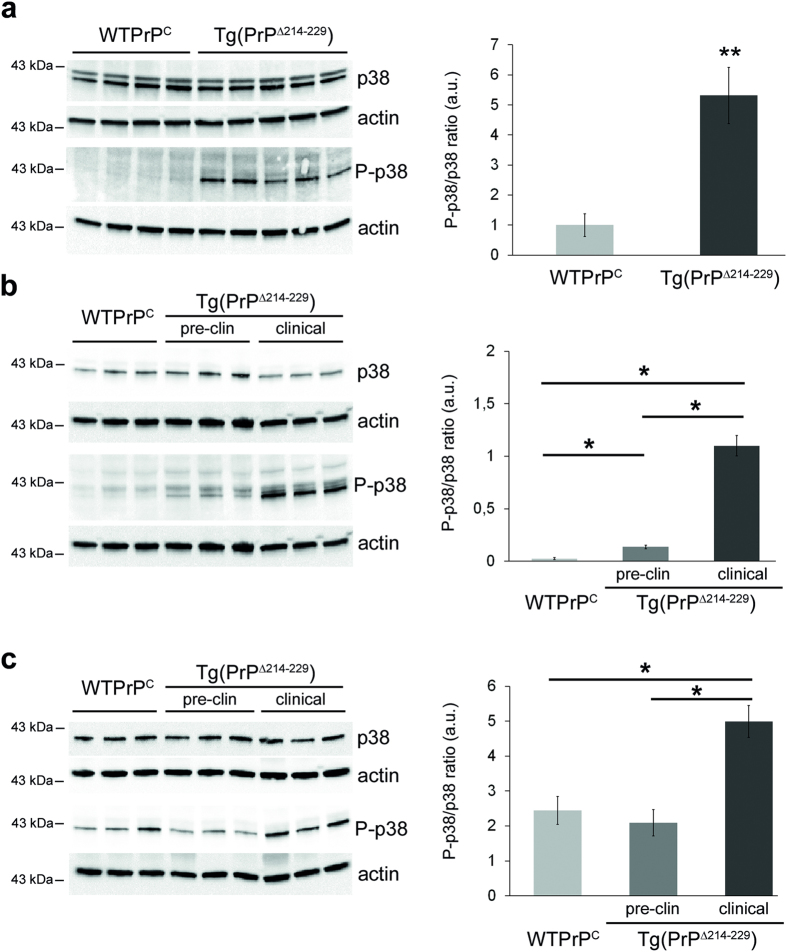
p38-MAPK is activated in brains of terminally-diseased and preclinical Tg(PrPΔ214–229) mice. (**a**) Western blots of forebrain homogenates from age-matched Tg(WTPrP^C^) (n = 4) and terminally-diseased Tg(PrPΔ214–229) mice (n = 5). Levels of total p38-MAPK are unchanged, whereas P-p38-MAPK (T180/Y182) is increased. Actin is used as a loading control. For densitometric analysis, intensities of P-p38-MAPK and p38-MAPK signals were first referred to their corresponding actin and then the ratio P-p38-MAPK to p38-MAPK was calculated. (**b**) Western blots of forebrain homogenates from age-matched Tg(WTPrP^C^) and Tg(PrPΔ214–229) mice either at a preclinical time point (17 weeks of age) or in clinical mice. The ratio P-p38/p38 was calculated as described before. (**c**) Western blots of cerebellar homogenates of Tg(WTPrP^C^) and Tg(PrPΔ214–229) mice at time points described in (**b**). Error bars show SEM (*p*-values of Student´s *t*-test are given in the main text). **p < 0.005; *p < 0.05.

The fact that PrPΔ214–229 is retained at the biosynthetic pathway and sensitive to EndoH digestion, prompted us to investigate cell death pathways linked to ER stress. We could not detect induction of UPR signaling pathways (upregulation of CHOP, increase in cleaved ATF6, phosphorylated eIF2a) in Tg(PrPΔ214–229) mice (Supplementary Fig. 4). Strikingly, we observed a clear increase of phosphorylated p38-MAPK (P-p38-MAPK) in the forebrain at clinical and pre-clinical time points (*t*-test: WTPrP^C^ versus clinical Tg(PrPΔ214–229) forebrain, *p* = 0.006; WTPrP^C^ versus clinical Tg(PrPΔ214–229), *p* = 0.0083) and in cerebellum at clinical time points (WTPrP^C^ versus clinical Tg(PrPΔ214–229), *p* = 0.026) (Fig. 5). Other putative prion-associated signaling pathways such as phosphorylation of Fyn^29^,^30^, JNK, ERK, STEP, c-PLA2^31^,^32^,^33^ or associated to prion-driven calcium disturbances^34^ such as calpain, were unaltered (Supplementary Fig. 5a–d and Supplementary Fig. 6a–c).

### PrPΔ214–229 is partially proteinase K (PK) resistant and accelerates disease in syngeneic hosts

**Figure 6:**
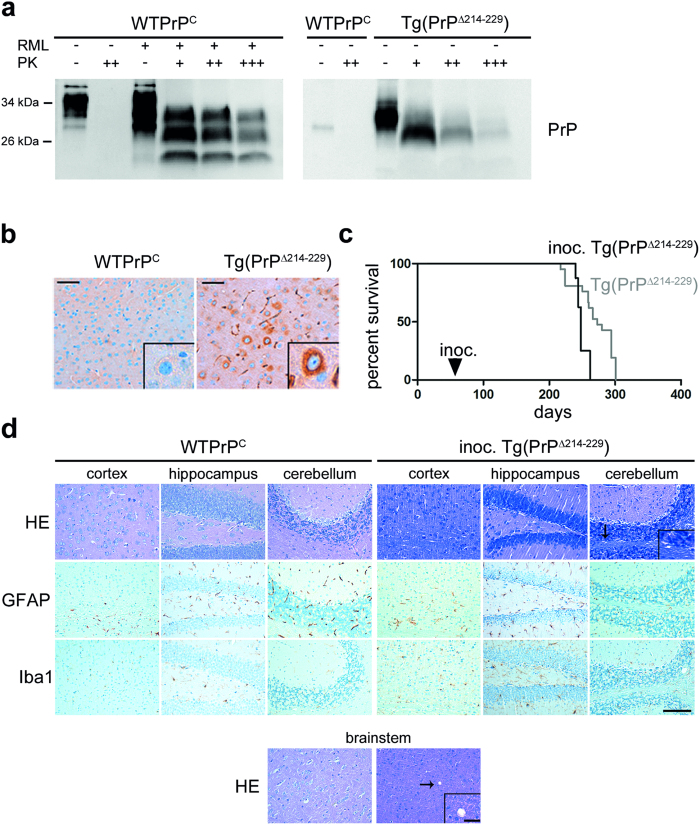
PrPΔ214–229 is partially PK resistant and accelerates disease in syngeneic host. (**a**) Western blot analyses of PrP in brain homogenates after PK digestion. 4 μl of 10% total brain homogenate were treated with increasing amounts of PK (10 μg/ml (+); 20 μg/ml (++) and 40 μg/ml (+++)). A wild type prion diseased mouse (inoculated with RML prions) was used as a positive control. WTPrP^C^ was detected with the POM1 antibody whereas Tg(PrPΔ214–229) was detected with the 3F4 antibody and therefore only unspecific bands are observed for WTPrP^C^ in this blot. Note that Tg(PrPΔ214–229) is PK resistant at the concentration widely used to define PrP^Sc^ (20 μg/ml), but compared to the prion diseased mice brain is less resistant at higher concentrations of PK. (**b**) Representative immunohistochemistry of cortical mouse brain sections stained with SAF84 antibody after PK digestion. Higher magnification shows accumulation of PK-resistant PrP surrounding the nucleus in Tg(PrPΔ214–229). Scale bar in (**b**,**d**) is 100 μm. (**c**) Kaplan-Meier survival curve of Tg(PrPΔ214–229) (grey) and of Tg(PrPΔ214–229) inoculated with brain homogenates obtained from clinically diseased Tg(PrPΔ214–229) mice (black). A significant acceleration of the disease is observed (Breslow test: p* = 0.007). (**d**) Brains from Tg(PrPΔ214–229) mice inoculated with Tg(PrPΔ214–229) brain homogenates (inoc. Tg(PrPΔ214–229)) presented very faint spongiosis (as seen with HE staining) mainly in cerebellum and striatum and no overt astro-/microgliosis (as seen with immunohistochemistry for GFAP and Iba1). Scale bar in the magnified picture is 25 μm.

When PrP is mutated and retained in the ER, it adopts features similar to PrP^Sc^ such as partial PK resistance and insolubility in non-ionic detergents^28^. Thus, we assessed PK resistance at a concentration widely used to define PrP^Sc^ (Fig. 6). PrPΔ214–229 from brain homogenates was partially resistant to PK digestion yet transmission experiments into highly prion-sensitive *tg*a20 indicator mice (n = 12) did not show detectable titers of prion infectivity when inoculated with pre-clinical (42 days old; n = 1) or clinical (280 days old; n = 2) brain homogenates. All *tg*a20 mice remained healthy >250 days post inoculation.

However, inoculation of brain homogenates from clinically sick Tg(PrPΔ214–229) mice into eight weeks old syngeneic hosts led to a significant acceleration of disease, with inoculated Tg(PrPΔ214–229) mice coming down with disease at 246.75 ± 1.06 days when compared to naive Tg(PrPΔ214–229) mice (270.28 ± 38.61 days, Breslow (Generalized Wilcoxon) test *p = 0.007; Fig. 6c). This acceleration of disease was not accompanied by gross neuropathological changes, although a slight vacuolation could be observed (Fig. 6d).

### ER stress leads to PrPΔ214–229 retention in the Golgi apparatus

**Figure 7:**
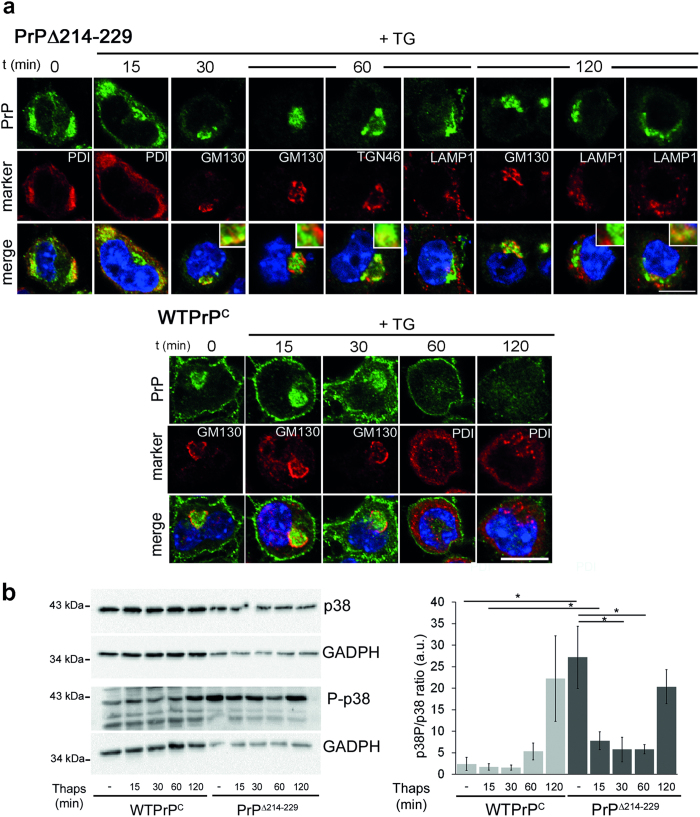
PrPΔ214–229 is retained in the Golgi after Thapsigargin treatment and p38-MAPK phosphorylation is increased. (**a**) N2a cells were treated with Thapsigargin (TG) for 10 min and fixed at different time points. PrPΔ214–229 (green) is found mainly in the ER (colocalizing with PDI; red) at steady-state but after 30 min it is found in the cis-Golgi (colocalizing with GM130; red) traveling to the trans-Golgi network (colocalizing with TGN46; red) after 60 min. At 120 min PrPΔ214–229 is found mainly colocalizing with GM130 again. At this time point, some degree of colocalization (inset) of PrPΔ214–229 with the lyosomal marker LAMP1 (red) could be observed. In steady-state conditions WTPrP^C^ is mainly found in the Golgi (colocalizing with GM130; red) and at the plasma membrane. At 60 and 120 min after TG treatment the intensity of the staining is decreased and WTPrP^C^ is found at the plasma membrane but not in Golgi anymore. Scale bar is 10 μm. (**b**) Representative Western blot showing that, at steady-state, there is a high level of phosphorylation of p38-MAPK in PrPΔ214–229 compared to WTPrP^C^ that decreases between 15–60 min to increase again at 120 min (*p < 0.05).

The fact that PrPΔ214–229 is retained in the ER but also partially detected at the plasma membrane is reminiscent of the recently described RESET pathway^19^. Thus, we investigated the trafficking of PrPΔ214–229 under stress conditions in N2a cells (Fig. 7).

At 0 and 15 min after Thapsigargin (TG) treatment, WTPrP^C^ was found in the Golgi and at the plasma membrane, whereas at 60 and 120 min after TG, WTPrP^C^ presented a diffuse intracellular and plasma membrane pattern. For PrPΔ214–229 we could observe that from 15–60 min after TG application, it moves from ER to the cis- and trans-Golgi to finally come back to the cis-Golgi after 120 min. Thus, although PrPΔ214–229 undergoes the RESET pathway under stress conditions, it does not follow a canonical RESET pathway, with almost no co-localization with the lysosomal marker LAMP-1 (Fig. 7a). As shown in Fig. 7b, PrPΔ214–229 presented with an elevated steady-state of P-p38-MAPK compared to WTPrP^C^ (*t*-test, *p* = 0,028) that unexpectedly was significantly decreased after 30 min (*t*-test, *p* = 0,050) and 60 min (*t*-test, *p* = 0,042) of stress induction to later increase again at 120 min (Fig. 7b). Interestingly, the decrease in P-p38-MAPK coincided with the passage of PrPΔ214–229 from the ER to the Golgi.

## Discussion

Here we investigated the intracellular trafficking and the associated signaling pathways of misfolded PrP^C^ partially lacking its C-terminus (PrPΔ214–229). Our findings in neuronal cell lines show that PrPΔ214–229 is devoid of complex glycans and retained in the ER. When expressed in mice lacking endogenous PrP^C^, PrPΔ214–229 leads to a fatal neurological disease which can be partially rescued by genetic reintroduction of PrP^C^. PrPΔ214–229 does not behave as a *bona fide* prion yet holds PrP^Sc^-like properties and leads to disease acceleration in syngeneic hosts. Neuronal loss in Tg(PrPΔ214–229) mice is accompanied by activation of the p38-MAPK. Thus, we define a novel neurodegenerative pathway associated with defective intracellular PrP trafficking that may be relevant in prion diseases and other neurodegenerative protein misfolding disorders.

PrP is retained in the ER in a number of instances, e.g. when (i) its disulfide bond is disrupted^19^,^35^, (ii) octapeptide repetitions are inserted in the N-terminus^28^,^36^, (iii) disease causing point mutations are present at the C-terminus^13^,^14^ or (iv) GPI-anchorage is disturbed^17^. Consequences of ER retention are diverse and include retrotranslocation of potentially neurotoxic PrP to the cytosol^15^,^17^,^36^ or enhanced targeting to lysosomal degradation^18^.

In cultured cells, PrPΔ214–229 presents some features similar to the recently described RESET pathway^19^ because in steady state conditions it is mainly retained in the ER but also partially found at the plasma membrane. Under stress, PrPΔ214–229 reaches the TGN, but in contrast to the RESET pathway it cannot traffic further. RESET cargo receptors to lysosomes are only partially defined, with Tmp21 achieving the transport from the ER to the Golgi^19^. From our results, it might be speculated that PrPΔ214–229 is able to bind Tmp21, thus reaching the Golgi. However, due to its C-terminal deletion, the binding to other cargo receptors required for further transport to the plasma membrane might be compromised, resulting in Golgi retention. Importantly, PrP^C^ retention in the Golgi occurs in prion infected N2a cells^37^, where PrP^Sc^ impairs post-Golgi trafficking. More recently, Bouybayoune *et al.* showed that transgenic mice expressing PrP with a C-terminal mutation (PrPD177N/M128) homologous to the human mutation causing FFI, accumulate mutant PrP in the Golgi^38^. These observations, together with our study, indicate that accumulation in the secretory pathway either through misfolding caused by mutations or by conversion to PrP^Sc^ could be a common mechanism for neurodegeneration related to PrP.

When expressed in mice, ER retention of PrPΔ214–229 leads to neuronal death. We therefore expected activation of signaling pathways related to ERAD^39^, yet we could not find evidence for this. Since the induction of such pathways is spatiotemporally controlled, it could be that the temporal resolution of our experiment was not optimal^7^. Most importantly, we demonstrated a clear activation of p38-MAPK that correlates with neurodegeneration. p38-MAPK (a stress-activated protein kinase, SAPK) is activated, among other signals, under ER stress conditions^40^ and is related to cell death in neurodegenerative diseases^41^. To find out if this kinase participates in the RESET pathway, we investigated p38-MAPK phosphorylation in N2a cells expressing PrPΔ214–229. We observed an elevated steady-state level of P-p38-MAPK that, to our surprise, showed a transient decrease after stress induction. Thus, one can hypothesize that it is the presence of PrPΔ214–229 in the cis-Golgi after undergoing RESET pathway that activates a p38-MAPK pathway, eventually leading to cell death. In this respect, it is worthy to note that the KDEL receptor (KDEL-R), a cis-Golgi resident that recycles chaperones containing the KDEL retrieval motif back to the ER^42^,^43^ and a key regulatory signaling receptor in ER-to-Golgi trafficking^44^,^45^,^46^,^47^, can activate p38-MAPK under ER stress further deciding the fate of the cell^48^,^49^. Although we found a clustering of PrPΔ214–229 colocalizing with the KDEL chaperones in steady-state conditions, the exact mechanism by which accumulation of PrPΔ214–229 leads to p38-MAPK phosphorylation as well as the possible participation of KDEL receptor clearly require further studies.

Neurotoxic PrP106-126 and PrP^Sc^ can activate p38-MAPK possibly by binding to plasma membrane-bound PrP^C^^29^,^50^. Given that PrPΔ214–229 shares some characteristics reminiscent of PrP^Sc^, this may contribute to p38-MAPK activation in our model. Although this hypothesis cannot be ruled out, PrPΔ214–229 is present at the plasma membrane at only low amounts and reintroduction of PrP^C^, potentially serving as a binding and trafficking partner for PrPΔ214–229, does not aggravate but rather reduce the toxic effects. Thus, plasma membrane mediated p38-MAPK activation seems unlikely.

Partial rescue of neurotoxicity by co-expression of one *Prnp* allele has also been observed in transgenic mice expressing toxic N-terminal deletion mutants of PrP^36^. This argues in favor of a scenario where the putative functions of PrP^C^ are partially normalized in spite of PrPΔ214–229 retention in the ER. In the model presented here, it could be that PrP^C^ dimerizes with PrPΔ214–229 thus restoring correct trafficking and proteolytic processing^51^. PrP^C^ cleavage events have key roles in physiological and neurodegenerative conditions by both, reducing PrP^C^ at the neuronal surface and releasing soluble and neuroprotective PrP into the extracellular space^52^,^53^,^54^,^55^. Although we did not directly assess, whether PrPΔ214–229 is structurally able to undergo these cleavages, insufficient trafficking of PrPΔ214–229 to the plasma membrane (where shedding is thought to take place) is the likely reason for the absence of shedding. As a consequence, absence of neuroprotective shed PrP^C^ might contribute to the phenotype of cells and mice expressing PrPΔ214–229.

In a number of protein misfolding disorders, inoculation of brain homogenate from an affected animal to a disease-prone host leads to acceleration of disease^56^. We detected the presence of PK-resistant PrPΔ214–229 and disease acceleration by inoculation into syngeneic hosts with absence of prion titers. This implies that PrPΔ214–229 can follow template-folded conversion as it has been described for other proteins in Alzheimer’s disease, Parkinson’s disease and tauopathies.

In conclusion, we show that ER retention of a C-terminal PrP mutant leads to spontaneous neurodegeneration and neurological disease. On a molecular level, this is characterized by a trafficking defect leading to p38-MAPK activation. It will be interesting to investigate, whether activation of the p38-MAPK pathway represents a common mechanistic end stretch for other conformational dementias.

## Methods

### Ethics Statement

Animal experiments were approved by the *Behörde für Gesundheit und Verbraucherschutz* of the *Freie und Hansestadt Hamburg* (permit numbers 80/08, 38/07 and 84/13). Procedures were in accordance with the guidelines of the animal facility of the *University Medical Center Hamburg-Eppendorf* and in compliance with the *Guide for the Care and Use of Laboratory Animals.*

### cDNA constructs

cDNA containing the mouse *Prnp* open reading frame with the 3F4 mAb epitope tag in pcDNA3.1 ( + )/ Zeo was a gift from M. Groschup (Friedrich-Loeffler-Institut, Greifswald-Insel Riems, Germany). Mutagenesis to delete amino acids 214–229 of PrP^C^ (GenBank^TM^ accession number NP035300) was performed with the QuickChange Site-Directed Mutagenesis Kit (Stratagene, Agilent Technologies). Clones were verified by DNA sequencing.

### Stably and transiently transfected cell lines and drug treatment

N2a cells were maintained in Dulbecco’s modified Eagle’s medium high glucose with L-gutamine, supplemented with 10% fetal bovine serum (GE Healthcare Life Sciences) in a 5% CO_2_ incubator. The generation of stable cell lines and transfections were performed as previously described^57^. Thapsigargin (TG; 0.1 μM; Sigma-Aldrich) was applied for 10 min and cells were fixed at different time points. At least three independent experiments were performed for the TG treatment.

For transient transfections, SH-SY5Y cells were cultivated as described earlier^58^ and transfected with 1 μg DNA by using Lipofectamine Plus reagent (Life Technologies).

### Generation of PrPΔ214–229 mice

To insert the *PrP*Δ*214–229* construct into the half-genomic expression vector (mPrPHG)^59^, a PmlI restriction site was inserted with QuickChange Lightning (Stratagene).

PrP^C^ was excised from mPrPHG by AgeI and PmlI (Fast Digest, Fermentas; Thermo Fischer Technologies). PrPΔ214–229 was cut with AgeI and PmlI and ligated with the mPrPHG. For pronuclear injection the mPrPHG vector was cut with SalI and NotI. The pronuclear injection was performed at the Transgenic Mouse Facility (ZMNH, Hamburg). Positive animals were selected by PCR. Animals were backcrossed at least six generations to PrP^0/0^ in order to generate transgenic mice lacking endogenous PrP^C^. To generate the WTPrP^C^ mice, littermates not expressing the transgene were backcrossed with C57Bl6 mice following a similar breeding scheme.

### Primary neurons

P0–P2 mice were used. Briefly, hippocampi were dissected and collected in 10 mM glucose in PBS containing 0.5 mg/ml papain (Sigma-Aldrich) and 10 μg/ml DNAse (Roche Diagnostics). After 30 min incubation at 37 °C, samples were washed with plating medium (MEM 1X (Gibco), 20 mM glucose (Sigma), 10% Horse serum (PAA Laboratories) and 3% NaHCO_3_ 7.5% (Gibco)) and plated in 6-well plates containing Poly-L-Lys-pretreated coverslips and incubated at 37 °C in a 5% CO_2_ cell culture incubator. After 4 hours, the media was changed to Neurobasal A medium (Gibco) containing 2% of B27 serum, Glutamax (Gibco) and penicillin/streptomycin (PAA Laboratories). Next day, AraC (Sigma-Aldrich) was added in order to kill any proliferating cells. Half of the media was changed every 3 days. Analysis of proteolytic PrP^C^ shedding in primary neurons was performed as described earlier^24^.

### Western blot analysis

Confluent cells on a 6-well plate or mouse brain samples were homogenized using RIPA buffer (200 μl for cells or 10% homogenate for tissue) containing a cocktail of protease inhibitors (CompleteTablets EDTA-free) and phosphatase inhibitors (PhosStop) (Roche) and processed for Western blot^24^. For PK digestion, the experimental procedure was performed as described before^24^ but using different amounts of PK (10 μg/ml; 20 μg/ml; 40 μg/ml).

### Confocal microscopy

Primary neurons were maintained in culture for one week before being processed for microscopy. Cells were fixed with 4% paraformaldehyde. After washing, cells were permeabilized with 0.1% Triton X-100 in PBS containing 0.3% of bovine serum albumin (BSA). Primary antibodies were diluted in 0.3% BSA/PBS and incubated for 1 h at room temperature (RT). After washing with PBS, secondary antibodies AlexaFluor 488 or 555 (Life Technologies) were incubated for 1 h at 1:500 diluted in 0.3% BSA/PBS and after washes in PBS samples were mounted with Dapi Fluoromount G (SouthernBiotech, Birmingham AL). For non-permeabilizing conditions, cells were washed with cold PBS and incubated with the primary antibody for 20 min on ice. Afterwards they were fixed with 4% paraformaldehyde and the protocol was followed as described above. Images were taken with a Laser Scanning Confocal Microscope TCS SP5 (Leica) and further analyzed with Leica Application Suite X.

### PNGase F and EndoH assay

PNGase F assay was performed as described before^24^. For the EndoH assay (New England BioLabs), brain samples (40 μg of protein) were mixed with 10X denaturing buffer to a final volume of 20 μl and heated for 10 min at 100 °C. 6 μl of Endo H was added to 10X the reaction buffer in a final volume of 40 μl. The reaction was incubated at 37 °C for 1 h.

### Lipid raft isolation

Lipid rafts were isolated as previously described^57^.

### Triton X-114 assay

The assay was performed as previously described^27^. Briefly, frontal cortex samples were homogenized in 10 vol. of 0.32 M sucrose in 50 mM HEPES/NaOH pH 7.4, centrifuged for 15 min at 8.000 *g* and the supernatant was further centrifuged at 26.000 *g* for 2 h. The resulting pellet was resuspended in H buffer (10 mM HEPES/NaOH pH 7.4) with the addition of 2% of precondensed Triton X-114 (Sigma-Aldrich) in a total volume of 200 μl (final concentration of 2 μg/μl). Samples were vortex mixed for 1–2 sec, let on ice for 5 min and centrifuged again at 8.880 *g* at 4 °C in a fixed angle rotor. The resulting pellet was washed with 0.2 ml of H buffer, centrifuged at 8.800 *g* for 10 min at 4 °C and the resulting pellet resuspended in 180 μl of H buffer. This was kept as the Insoluble Pellet. The supernatant was layered over a 0.3 ml of 6% sucrose cushion in T buffer (10 mM Tris HCl pH 7.4, 0.15 M NaCl) and 0.06% precondensed Triton X-114, incubated at 30 °C for 3 min and further centrifuged at 3000 *g* for 3 min in a swinging bucket rotor. The sucrose cushion was removed from the pellet (detergent phase) which was resuspended in 180 μl of H buffer.

The supernatant (upper aqueous phase) was mixed with 0.5%(v/v) precondensed Triton X-114, vortexed 1–2 sec, kept 5 min on ice, and further layered over a 0.3 ml 6% sucrose cushion in T buffer, incubated at 30 °C for 3 min and centrifuged again at 3000 *g* for 3 min in a swinging bucket rotor. The pellet was discarded and the upper phase mixed again with 2% (v/v) precondensed Triton X-114 and processed again as described in the step before but without the sucrose cushion. After the last centrifugation, the supernatant was kept as final aqueous phase. An equal amount of sample was then mixed with 4× loading buffer and 30 μl of sample was subjected to gel electrophoresis and Western blot.

### Solubility assay

The assay was performed as described earlier^60^. Cells were washed with cold PBS, scraped off the plate and lysed with cold Buffer A (0.5% Triton X-100 and 0.5% sodium deoxycholic acid in PBS) containing a cocktail of protease inhibitors (Roche). After 10 min incubation on ice, samples were centrifuged at 15.000 *g* for 20 min at 4 °C. Supernatant was mixed with 4× loading buffer and the pellet was washed once in PBS, incubated in 1% SDS plus benzonase (Sigma) for 10 min at room temperature. Samples were mixed with 4× loading buffer and boiled for 5 min at 95 °C. Equal amounts of sample were subjected to gel electrophoresis and Western blot.

### Immunohistochemistry

Sections were stained using a Ventana Benchmark XT (Ventana). Deparaffinised sections were incubated for 30–60 min in CC1 solution (Ventana) for antigen retrieval. Primary antibodies were diluted in 5% goat serum (Dianova), 45% Tris-buffered saline pH 7.6 (TBS) and 0, 1% Triton X-100 in antibody diluent solution (Zytomed). Sections were then incubated with primary antibody for 1 h (see supplemental list). Anti-mouse histofine Simple Stain MAX PO Universal immunoperoxidase polymer (Nichirei Biosciences) were used as secondary antibody. Detection of secondary antibodies was performed with an ultraview universal DAB detection kit from Ventana with appropriate counterstaining and sections were cover-slipped using TissueTek glove mounting media (Sakura Finetek). To allow comparability for each analysis, sections of Tg(PrPΔ214–229) mice and controls were stained in one machine run.

For PrP^Sc^ staining, samples were treated for 30 min in Peroxidase-Blocking buffer (0, 3% H_2_O_2_) and 30 min in 98% formic acid. After washing with water, samples were autoclaved for 5 min at 121 °C in citrate buffer pH 6. The protocol was then followed as described above with the additional step of PK digestion followed by incubation of SAF84 antibody.

### Statistical analysis

For statistical analysis, IBM SPSS Statistics 22 and GraphPad Prism 5 statistic software programs were used. In order to compare differences between Kaplan-Meier survival curves, the Breslow test was used. For comparison between the groups in Western blots and neuronal countings, Student’s *t-*test was used. Statistical significance was considered when *p*-values were as follows: *p < 0.05, **p < 0.005, ***p < 0.001.

## Additional Information

**How to cite this article**: Puig, B. *et al.* Secretory pathway retention of mutant prion protein induces p38-MAPK activation and lethal disease in mice. *Sci. Rep.* **6**, 24970; doi: 10.1038/srep24970 (2016).

## Supplementary Material

Supplementary Information
