# Supplementary material for: Secretory pathway retention of mutant prion protein induces p38-MAPK activation and lethal disease in mice
Source: Sci Rep. 2016 Apr 27;6:24970. doi: 10.1038/srep24970 (PMC4847012; doi:10.1038/srep24970)
Supplement: Supplementary Information [file srep24970-s1.pdf]

Supplementary information for "Secretory pathway retention of mutant prion protein induces p38-MAPK activation and lethal disease in mice"

Berta Puig, Hermann C. Altmeppen, Sarah Ulbrich, Luise Linsenmeier, Susanne Krasemann, Karima Chakroun, Claudia Y. Acevedo-Morantes, Holger Wille, Jörg Tatzelt, and Markus Glatzel

Supplementary Fig. 1. PrPΔ214-229 is Endo H sensitive and GPI-anchored in SHSY5Y cells. (a) Lysates of SHSY5Y cells transiently transfected with WTPrP^{C} or PrPΔ214-229 were treated with EndoH. PrPΔ214-229 is EndoH sensitive, thus confirming the results obtained with the N2a cells. (b) After treating SHSY5Y cells transiently transfected with WTPrP^{C} and PrPΔ214-229 with Triton X-100, PrPΔ214-229 is localized at the pellet fraction, indicative of misfolding, as obtained with N2a cells (Fig. 1). (c) Confocal images of SHSY5Y cells transiently transfected with WTPrP^{C} or PrPΔ214-229 and labeled for PrP (green) show intracellular retention of PrPΔ214-229, presumably in the ER, under permeabilized conditions. Under non-permeabilizing conditions there is decreased presence of PrPΔ214-229 at the plasma membrane as observed for stably transfected N2a cells. Scale bar is 10 μm.

Supplementary Fig. 2. Characterization of a low expressing PrPΔ214-229 line of transgenic mice. The transgenic mouse line Tg((PrPΔ214-229)L52) shows approximately 12 % of WTPrP^{C} expression that is set to 100%.

Supplementary Fig. 3. Tg(PrPΔ214-229) mice lose weight around week 30 and brains present with little vacuolation in the white matter of the cerebellum, PK resistant PrP and no signs of gliosis. (a) Animals were weighted once a week. Starting at week 30, Tg(PrPΔ214-229) mice gradually lose weight. (b) Hematoxylin-eosin staining shows slight vacuolation in the white matter of the cerebellum (arrows). Magnification shows also vacuoles in the white matter. Cortex and hippocampus are the areas where PK resistant PrP is found as revealed with the SAF84 antibody after PK digestion (arrow point to intracellular aggregates). No signs of gliosis are seen in any of the observed areas as revealed with GFAP staining (astrocytes) and with Iba1 staining (microglia). Scale bar is 100 μm.

Supplementary Fig. 4. No canonical ER stress is observed in Tg(PrPΔ214-229) mouse brains. Representative Western blots of brain homogenates from clinical Tg(PrPΔ214-229) mice compared to age-matched WTPrP^C mice or from pre-clinical Tg(PrPΔ214-229) mice (17 weeks old) developed with antibodies against typical ER stress markers Grp78 (Bip) (a), eIF2α and activated eIF2α (b), ATF-6 and CHOP (c). There are no significant differences either at pre-clinical or clinical time points.

Supplementary Fig. 5. Fyn, ERK 1/2 and SAPK/JNK are not activate in Tg(PrPΔ214-229) mouse brains. Representative Western blots of brain homogenates from clinical Tg(PrPΔ214-229) (n=5) compared to age-matched WTPrP^C mice (n=4) of: (a) Src kinase (when it is not phosphorylated at position Y416 (Src nonY416) or when it is active and phosphorylated at position Y416 (SrcY416). (b) Fyn kinase when it is phosphorylated at position Y528 (FynY528). Blots were first related to actin and then to total Fyn. No differences were seen between WTPrP^C and PrPΔ214-229. Also, no differences were observed for phosphorylated ERK 1/2 (c) and for phosphorylated SAPK/JNK (d). Tubulin blots for total Fyn and total ERK1/2 are the same because the blot was re-used without stripping due to different sizes of the proteins of interest.

Supplementary Fig. 6. Other signaling proteins described as being activated through PrP are not activated in Tg(PrPΔ214-229) mice. Western blots of (a) STEP, (b) cPLA2 and (c) calpain showed no differences between WTPrP^C and Tg(PrPΔ214-229) brain homogenates.

Supplementary Fig. 1

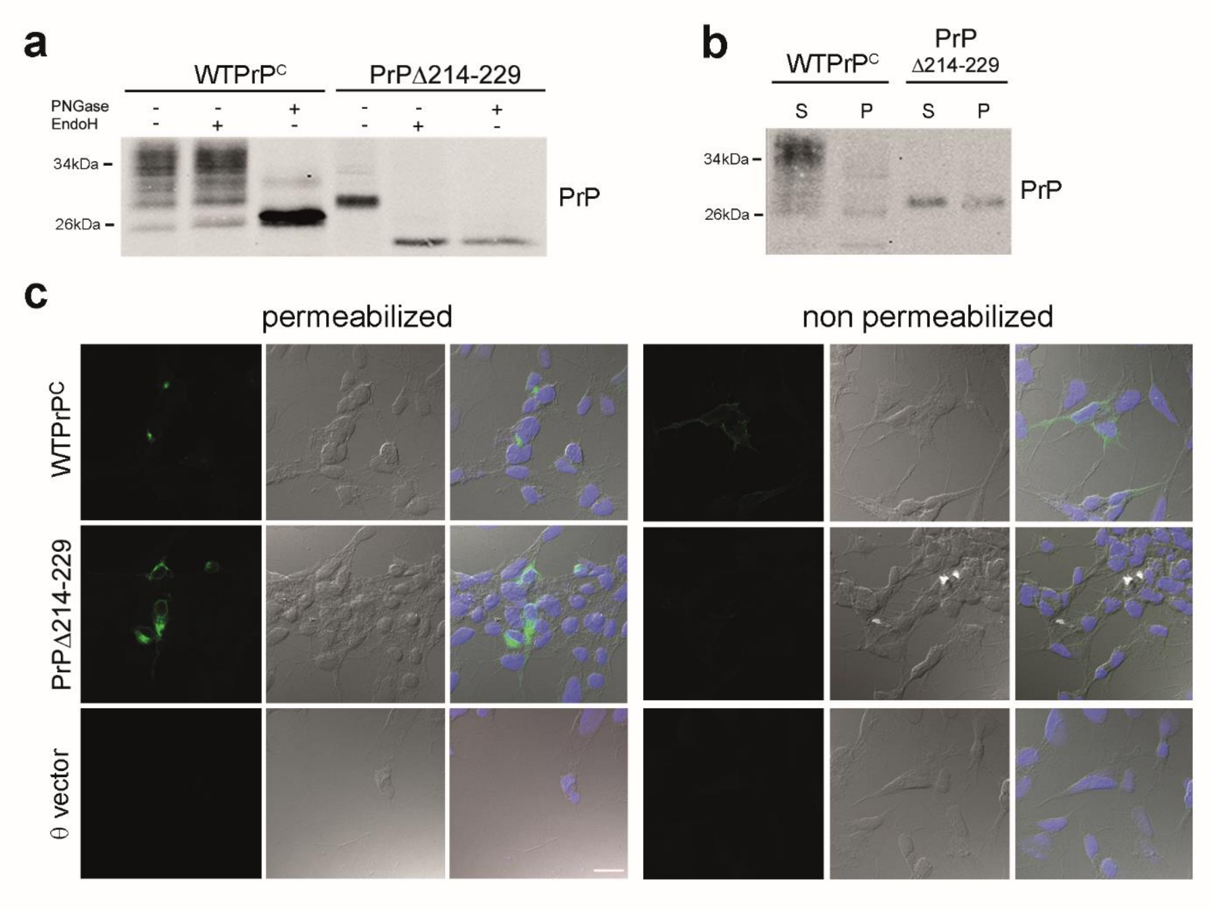

Supplementary Fig. 2

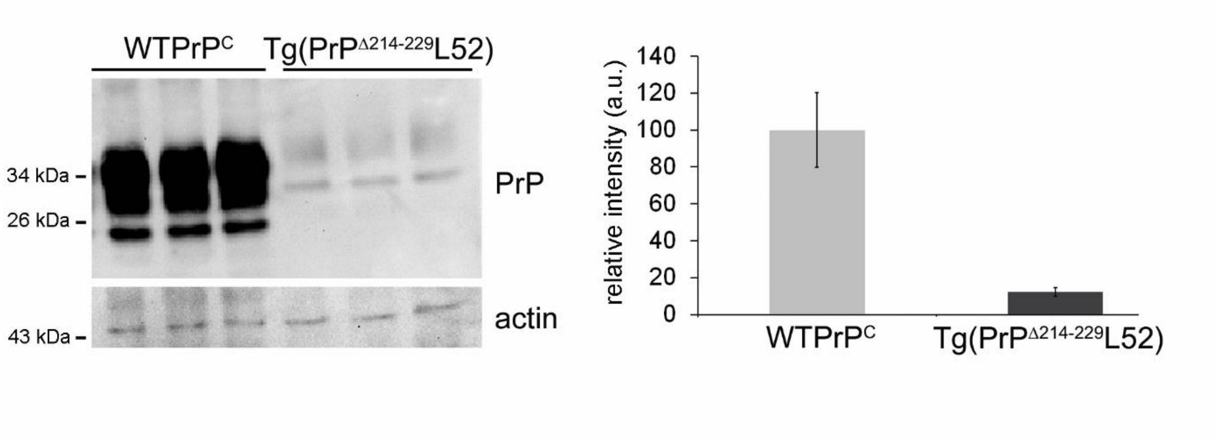

Supplementary Fig. 3

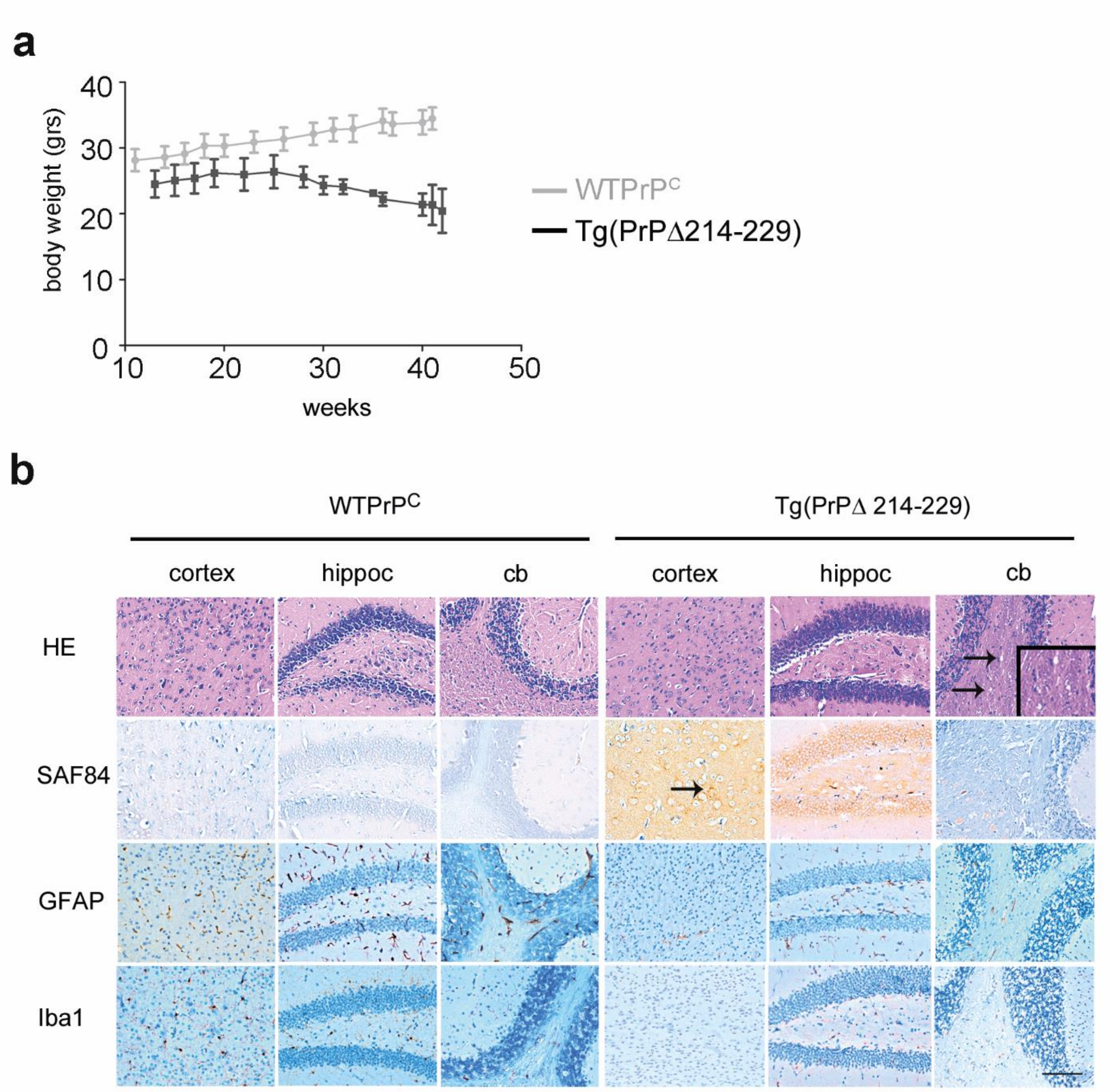

Supplementary Fig. 4

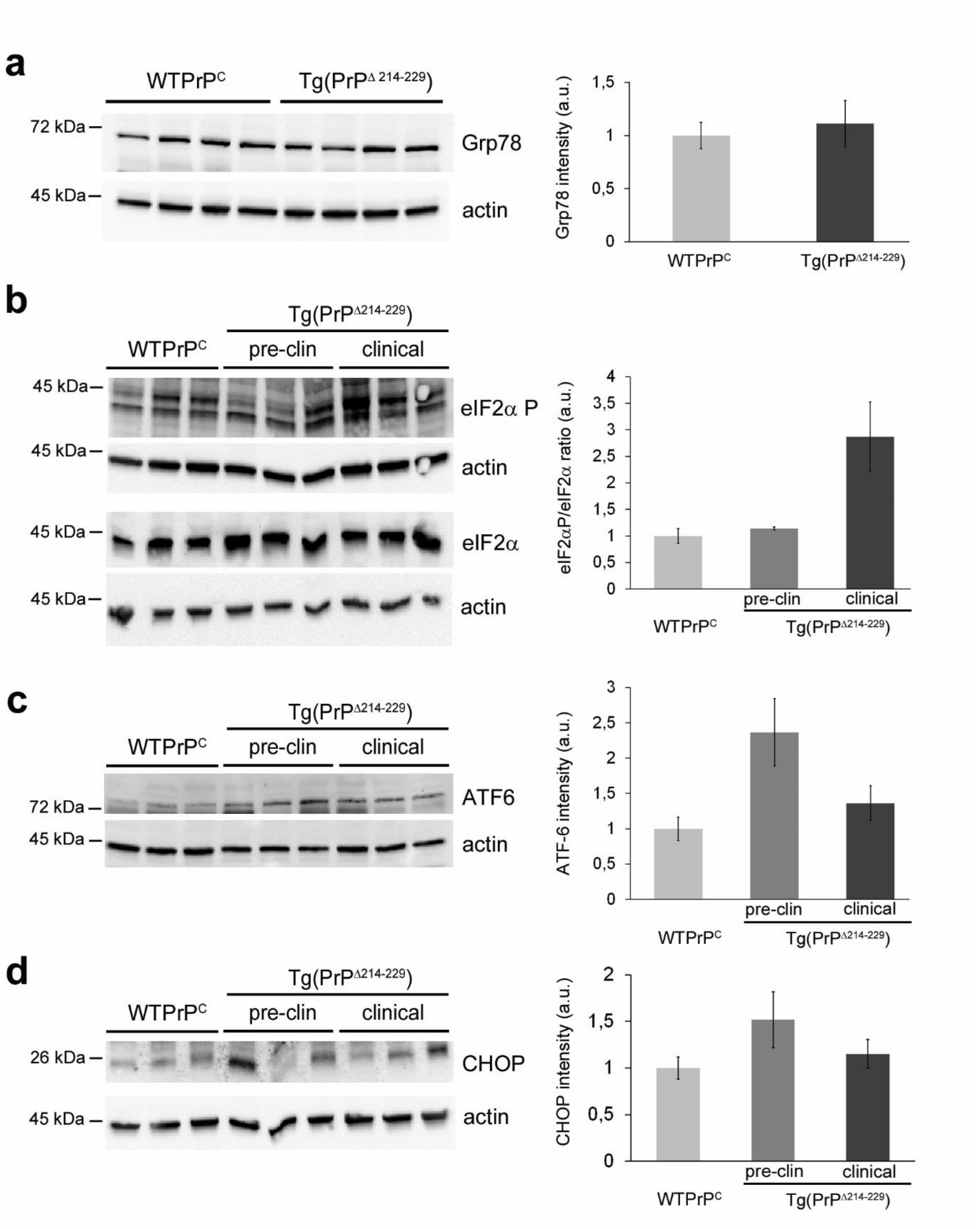

Supplementary Fig. 5

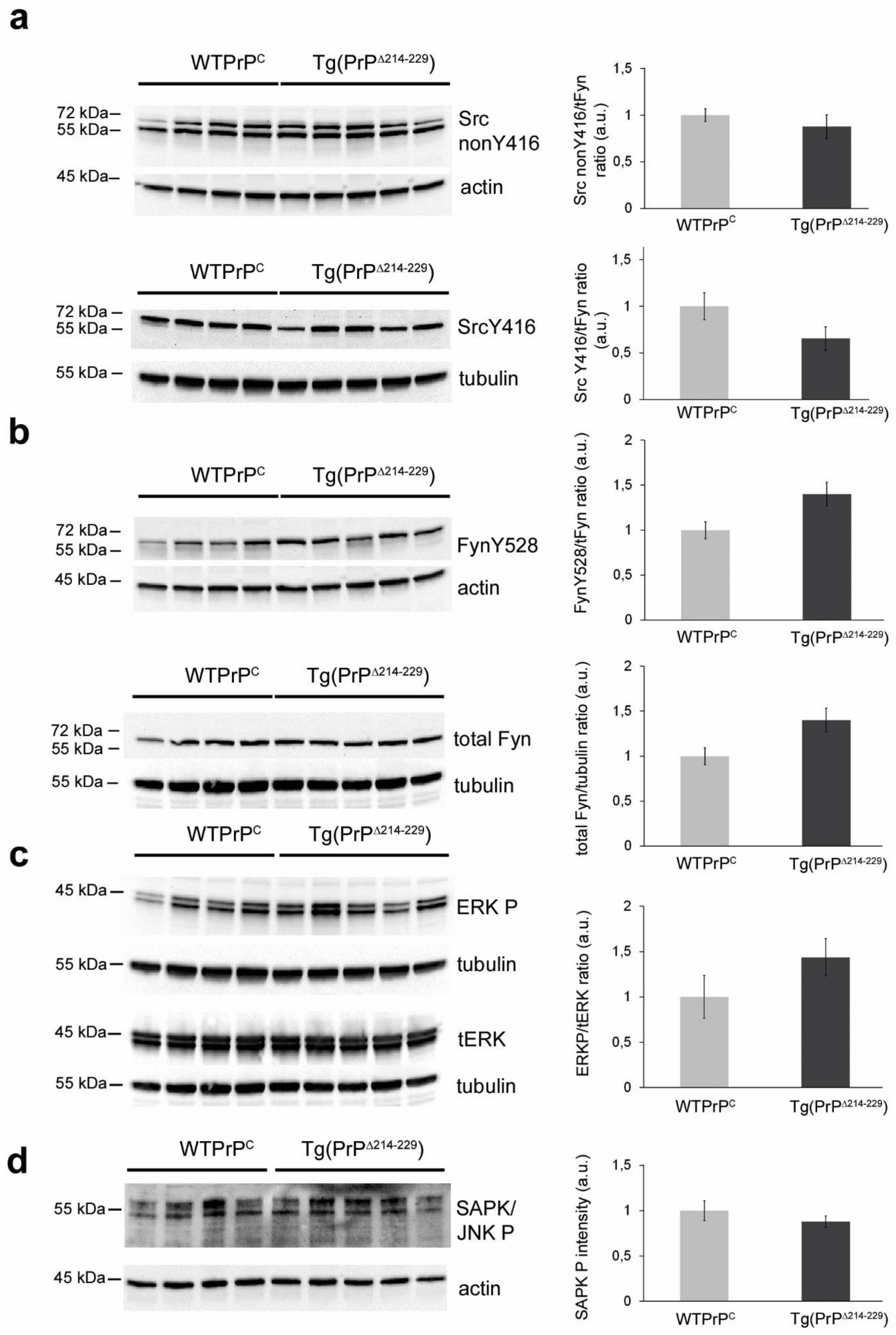

Supplementary Fig. 6

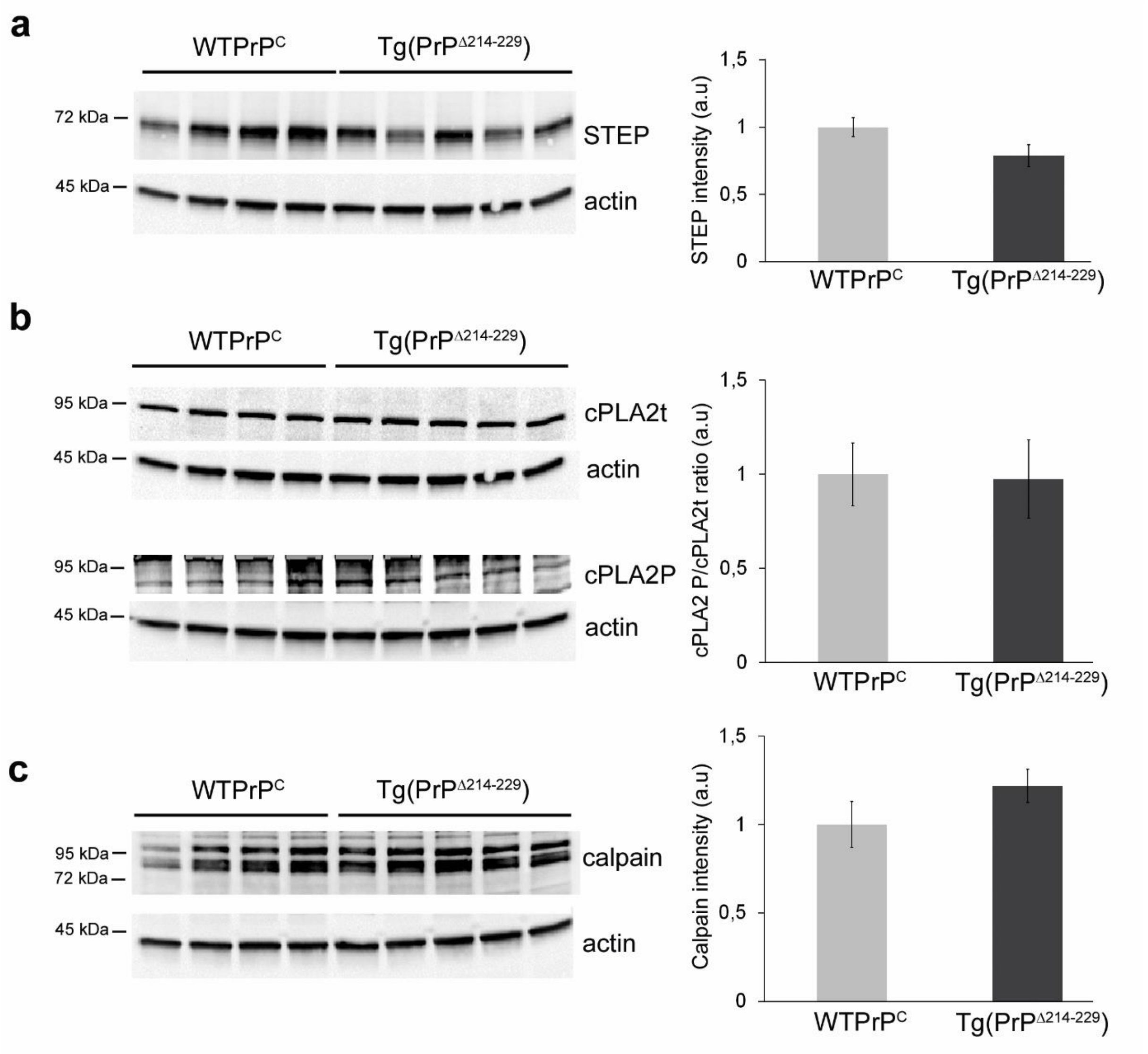

Supplementary Methods

Generation of PrPΔ214-229

Primers used for deletion from amino acid 214-229: F: GGTGGAGCAGATGATGTGCTCCAGCAGCACC

GTGC and R: GCACGGTGCTGCTGGAGCACATCTGCTCCACC.

Generation of Tg(PrPΔ214-229) mice

To insert the PmlI restriction site after the stop codon of PrPΔ214-229 DNA the following primers were used: F: CCCAAGGAGAAA**CACGTG**CCCTCGAGGTCCTTC; R: GAAGGACCTCGAGGG**CACGTG**TTTCTCCTTGGG (PmlI restriction site is in bold). Positive animals for the transgene were selected by PCR using the following primers: F: ATGGCGAACCTTGGCTACTG; R: GCTGCTGGAGCACATCTGCT.

Table 1- List of antibodies and dilutions used in this study

Antibody	Use	Dilution	Source
**3F4 (PrP)**	WB<br>ICC	1:1000<br>1:200	Covance
**POM 1 (PrP)**	WB<br>IHC	1:2000<br>1:250	Prof.A. Aguzzi (Zurich)
**POM 2 (PrP)**	WB	1:2000	Prof.A. Aguzzi (Zurich)
**Calnexin**	ICC	1:300	Stressgene
**Actin**	WB	1:2000	Millipore
**Tubulin**	WB	1:5000	Dev. Stud. Hybridoma Bank, Univ. of Iowa
**Src Family (Y416)**	WB	1:1000	Cell Signaling
**Src Family (non Y416)**	WB	1:1000	Cell Signaling
**Total Src Family**	WB	1:1000	Cell Signaling
**Fyn Y518**	WB	1:1000	BD Transduction Laboratories
**Fyn**	WB	1:1000	Cell Signaling
**p38 MAPK**	WB	1:1000	Cell Signaling
**P-p38-MAPK T180/Y182**	WB	1:500	Cell Signaling
**p44/42 MAPK(ERK1/2)**	WB	1:1000	Cell Signaling
**P-p44/42(T202/Y204)**	WB	1:1000	Cell Signaling
**P-MSK1 (T71)**	WB	1:000	Cell Signaling
**P-HSP27 (S82)**	WB	1:1000	Cell Signaling
**p-ATF-2(T71)**	WB	1:1000	Cell Signaling
**Calpain**	WB	1:1000	Cell Signaling
**eIF2alpha**	WB	1:1000	Cell Signaling
**P-eiF2alpha (S51)**	WB	1:1000	Cell Signaling
**ATF-6**	WB	1:1000	Novus Biologicals
**GrP78**	WB	1:1000	BD Transduction Laboratories
**CHOP**	WB	1:500	Cell Signaling
**cPLA2**	WB	1:1000	Cell Signaling
**P-cPLA2 (S505)**	WB	1:1000	Cell Signaling
**Beta III tubulin**	ICC	1:500	Sigma Aldrich
**PDI**	ICC	1:400	StressMarq Biosciences Inc.
**GM130**	ICC	1:300	Abcam
**LAMP-1**	ICC	1:250	Clone 1D4B Dev. Stud. Hybridoma Bank, Univ. of Iowa
**TGN46**	ICC	1:500	Abcam
**KDEL**	ICC	1:400	Pierce (Thermo Scientific)
**KDEL Receptor**	ICC	1:400	Abcam
**Iba-1**	IHC	1:500	Wako Chemicals
**GFAP**	IHC	1:200	DAKO
**SAF 84**	IHC	1:100	Cayman Chemicals
